# 30 years of ENLP: developing nutrition leaders for positive change – lessons learnt and ways forward

**DOI:** 10.1017/jns.2026.10092

**Published:** 2026-07-09

**Authors:** Nevena Ivanović, Sara Dobani, Holly R. Neill, Richard J. Webb, Alison M. Gallagher

**Affiliations:** 1 Faculty of Pharmacy, Department of Bromatology, University of Belgrade, Belgrade, Serbia; 2 Human Nutrition Unit, Department of Food and Drug, University of Parma, Italy; 3 Yakult UK & Ireland Ltd, UK; 4 Faculty of Human and Digital Sciences, Liverpool Hope University, UK; 5 School of Biomedical Sciences, Ulster University, UK

**Keywords:** Alumni network, Enhancing leadership, European Nutrition Leadership Programme, Food and nutrition professionals, Leadership development

## Abstract

Launched in 1994, the European Nutrition Leadership Platform (ENLP) has evolved over three decades into a unique and internationally recognised initiative for the development of leadership skills in the field of nutrition and public health. Through its two training programmes, the ENLP Essentials programme, designed primarily for early-career professionals and emerging leaders in nutrition, food science, and health, and the ENLP Advanced programme, tailored to mid-career and senior professionals seeking to enhance their leadership capacity, equips participants with scientific communication and interpersonal skills while fostering resilience, authenticity, and cross-sector collaboration. With more than 1000 graduates, many of whom now hold leading positions in academia, industry, government, and non-governmental organisations, the programme has proven its impact on professional development and the nutrition landscape. At IUNS-ICN 2025 in Paris, the ENLP celebrated its 30^th^ anniversary and emphasised that leadership in the nutrition sector is best defined by authenticity, caring, resilience, and shared values. In the coming decades, the programme will continue to focus on inclusivity, adaptability, and vision, demonstrating that investing in people is the most powerful lever for improving nutrition and public health.

## Introduction

The European Nutrition Leadership Platform (ENLP; https://enlp.eu.com), established in 1994 by leading nutrition experts, has evolved over three decades into an internationally recognised initiative. Its primary objective is to cultivate a new generation of nutrition leaders equipped with scientific expertise as well as leadership, communication, and interpersonal competencies required to drive meaningful change. The programme emphasises leadership development by providing participants with practical tools for teamwork, adaptive communication, conflict resolution, and the formulation of actionable personal development plans. Over the years, the ENLP has evolved with the changing landscape of nutrition and public health. What started as a one-week seminar in Luxembourg, organised by Wageningen University, has evolved into a two-track programme that continues to attract food and nutrition professionals from all sectors and consists of: (1) ENLP Essentials programme tailored for early-career professionals in nutrition and health; and (2) ENLP Advanced programme for experienced professionals who wish to deepen their leadership potential.

An international committee selects participants through a competitive application process. While the ENLP Essentials programme initially targeted final-year doctoral students and early-stage postdoctoral researchers, eligibility has broadened to include individuals with a master’s degree or relevant professional experience. In 2010, 16 years after the ENLP Essentials programme commenced, the ENLP Advanced programme was born. Driven by a strong wish from some of the then 450 ENLP alumni for a follow-up of the ENLP Essentials programme, a 3-day leadership training programme was designed to provide skills in the specific areas of most relevance to mid-career food, nutrition, and dietetic professionals. The curriculum of both programmes combines preparatory online activities with intensive face-to-face training, coaching, and reflective exercises in a collaborative environment. In addition to leadership development, ENLP programmes address contemporary challenges in nutrition and food science through leadership, effective communication, and collaborative team approaches. This approach enables participants to define their leadership style, clarify their values, and develop confidence to lead in diverse professional settings.

## A growing alumni network and global influence

**Figure 1.**
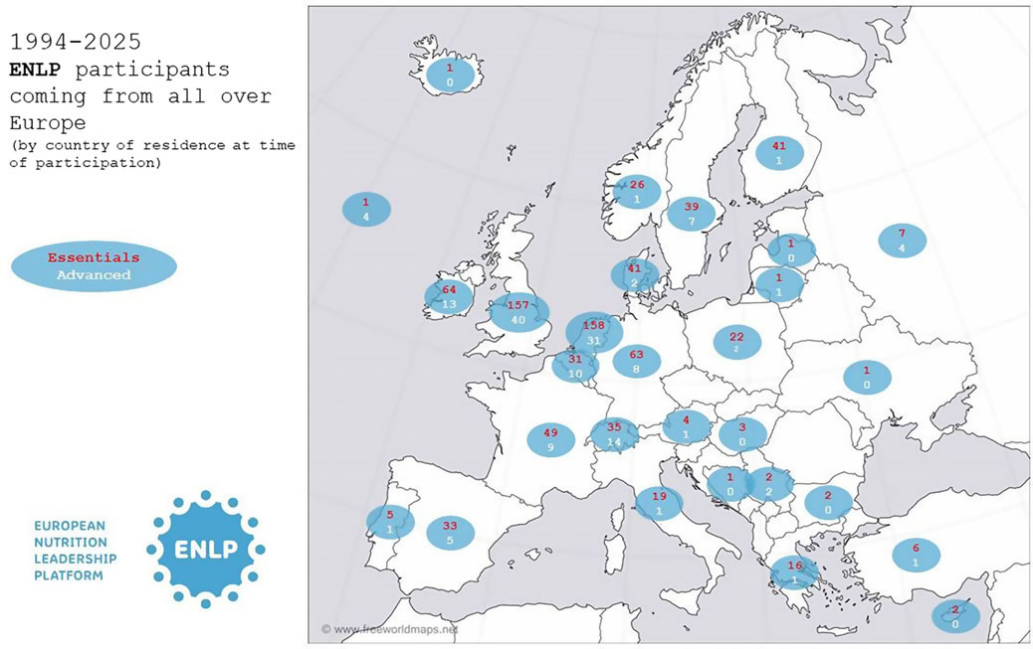
Geographical distribution of ENLP participants according to country of residence at the time of participation.

To date, over 1000 professionals from more than 230 organisations have participated in ENLP programmes. The geographical distribution of participants according to their country of residence at the time of participation is presented in Figure 1. Founded in the late 1990s, the ENLP Alumni continues to serve as a dynamic platform for lifelong professional development, mentoring, and collaboration. ENLP Alumni contribute to research, education, industrial innovation, and policy development across Europe and beyond. Besides the two annual training programmes (ENLP Essentials and ENLP Advanced), ENLP, through its ENLP Alumni also organises sessions in conferences, events, and ENLP Local Circles (which undertake regional activities); these activities serve to connect participants from the different years of the ENLP programmes, thereby nurturing and growing this engaged network. As highlighted in previous publications marking the 10^th^ and 15^th^ anniversaries of the programme,^(1,2)^ many former participants now hold leadership positions in academia, industry, public health, and government. In the alumni’s testimonials, the ENLP experience is repeatedly described as a turning point in their professional and often personal development.

In recent years, the network has embraced digital community tools and used the community management platform *Mobilize* as a central hub. In 2025, the platform has more than 550 active members and provides a space not only for networking alumni, but also for sharing job opportunities, calls for collaboration, events, and resources in various fields. The *Mobilize* management platform reflects the ENLP’s commitment to ongoing engagement and professional development beyond education itself.

## What makes the ENLP so unique?

The distinctive character of the ENLP reflects its intentional design to integrate leadership, communication, and interpersonal competencies with scientific expertise in nutrition and health. Alumni testimonials over the years have reaffirmed these principles, particularly highlighting the programme’s interactive learning environment, scientific relevance, and cross-sector engagement.

These defining elements are reflected in the programme’s structure and learning approach, which are characterised by: (a) interactive learning: participants engage in simulations, role-play, small group tasks and peer feedback, which enables in-depth personal reflection and development; (b) scientific relevance: leadership skills are developed in the context of nutrition and health sciences; and (c) cross-sectoral approach: the ENLP programmes bring together professionals from academia, government, non-governmental organisations and industry, fostering interdisciplinary collaboration.

## 30^th^ Anniversary celebrated at IUNS-ICN 2025 in Paris

As part of the 30^th^ anniversary celebrations, the ENLP hosted a Symposium Session, ‘30 Years of ENLP - Nurturing Nutrition Leadership for Positive Change: Lessons Learned and Future Directions’, at the 23^rd^ International Union of Nutritional Sciences International Congress on Nutrition (IUNS-ICN) 2025 in Paris. The symposium was chaired by Professor Alison Gallagher (Chair of the ENLP Board), Dr Holly Neill (ENLP Conference Team Member), and Ellie Hadjilucas (ENLP Board Member Secretariat). Following an introduction to the ENLP, video testimonials from ENLP alumni and promotion of the next round of applications, four ENLP alumni shared their personal experiences of effective leadership; namely, Professor Tom Hill (Newcastle University, UK), Dr Nicolas Gausserès (Danone, France), Professor Nevena Ivanović (University of Belgrade, Serbia), and Dr. Milka Sokolović (European Public Health Alliance, Belgium).

The four perspectives illustrated the diversity of leadership pathways shaped by the ENLP experience. Professor Tom Hill reflected on leadership as a journey of belief, humility, and vulnerability. He illustrated how belief, initially ‘borrowed’ from others, can develop into belief in oneself and others. Through lessons in listening, embracing uncertainty, and reframing failure, he showed that leadership is not defined by perfection but by continuous growth and the courage to evolve, creating space for others to thrive. Dr Nicolas Gausserès focused on the strategic dimension of leadership, shaped by unpredictability and change. Drawing on industry experience, he emphasised that leaders must anticipate uncertainty and act from clarity of values rather than fear, maintaining the freedom to say ‘no’ when necessary. He also reminded the audience that leaders shape their future through purposeful choices, openness, and sharing ideas, turning reflection into forward motion.

Through personal experiences in academia, parenting, and sport, Professor Nevena Ivanović described how leadership evolves from power and control into authentic connection and mutual growth. Using the metaphor of dragon boat paddling, she illustrated leadership as a collective rhythm built on listening, trust, and empathy, concluding that true leadership is often quiet yet deeply felt. Dr Milka Sokolović shared a powerful narrative of resilience, purpose, and transformation. She emphasised that her success was not achieved despite failure, but because of it. Through shared personal experiences and professional reinvention, she demonstrated that resilience is cultivated, not innate, and that meaningful leadership is grounded in purpose, empathy, and the ability to build loyal, trusting teams. For her, leadership lies not in titles but in the people who choose to follow. Together, these four perspectives showcased the diversity and depth of leadership within the ENLP community, uniting around a shared understanding that authenticity, resilience, reflection, and care form the essence of effective leadership.

Their reflections set the stage for the interactive Leadership Trait Card Activity that followed, inviting participants to explore their own traits through discussion and shared reflection. Participants were divided into small groups with each assigned a leadership trait related to the speakers’ presentations (vulnerability, supportive, reliable, or empathetic) and asked to discuss on groups ‘Why does this trait matter to you?’, ‘When have you seen it in action?’ and ‘What was the impact?’. Additional questions were provided to encourage further discussion and reflection, if required, including: ‘Who has shaped your leadership style the most and why?’, ‘What leadership lesson have you learned the hard way?’, ‘What is something you used to hide about yourself as a leader but now accept?’ and ‘What do you wish more people knew about leadership?’. These prompts facilitated in-depth discussion and encouraged participants to connect personal experiences with specific leadership traits. The group activities concluded with participants summarising their insights and identifying future directions for their leadership development. Mark Hollingsworth, Chief Executive at The Nutrition Society, shared his enthusiasm at gaining a greater insight to the ENLP values and transformative experience, highlighting how this ethos is universal and can translate across to other non-European leadership programmes. Professor Gallagher subsequently responded to a delegate query regarding the link between the ENLP and the African Nutrition Leadership Programme (ANLP), highlighting the hope and anticipation for future collaborations.

At the end of the symposium, speakers were asked to reflect on what ENLP has meant to them. They emphasised the acceptance of their own leadership style, the importance of resilience, the need for constant self-reflection, and the realisation that failure and vulnerability are an integral part of leadership. Finally, this symposium underlined the ENLP philosophy that leadership is not defined by titles, but by behaviour, authenticity, and shared values.

## Looking to the future: the next 30 years

Looking to the future, the ENLP remains committed to developing prudent, resilient, and value-oriented leaders in the food and nutrition sector. Emerging challenges such as climate change, food system sustainability, and the impact of the digital food landscape on eating habits require not only technical knowledge, but also vision, empathy, and cross-sector collaboration, including strengthened partnerships with other Nutrition Leadership Platforms (NLPs) worldwide.

Since the ENLP began, other Nutrition Leadership Platforms (NLPs) have been established in Africa (ANLP; http://www.africanutritionleadership.org/), South-East Asia (SEANLP; https://www.seameo-recfon.org/seanlp/), Latin America (https://lideresnutricion.blogspot.com/), and Oceania (ONLP; https://www.onlp.org/). Together, all these initiatives share a common understanding of leadership as the ability to ‘lead from where you stand’, grounded in team building, leadership training, effective communication, and teamwork. This global network creates opportunities for mutual learning, exchange of the best practices, and future joint initiatives.

The ENLP’s future priorities include: (1) expanding access to leadership training for all who want to develop and grow in this area, especially in low-income countries; (2) increasing the reach and engagement of the alumni network across Europe and beyond; (3) integrating new topics into the curriculum, such as the transformation of food systems, sustainable nutrition, the impact of the digital food landscape on eating habits, and new challenges in achieving food security.

The ENLP has consistently demonstrated that investment in human capital is essential for sustainable progress in nutrition and public health. This milestone highlights both the programme’s longstanding tradition and its significant potential for future impact.

## Conclusion

Over the past 30 years, the ENLP has built a strong legacy by empowering professionals to lead with integrity, empathy, and purpose. The ENLP 30^th^ anniversary symposium, held in Paris as part of IUNS-ICN 2025, clearly demonstrated that leadership in the food and nutrition sector requires behaviours and attitudes that foster self-confidence, resilience, self-reflection, and the ability to empower others. Through a combination of interactive training, mentoring, and the strength of its alumni network, the ENLP continues to prepare leaders to tackle the complex and evolving challenges in nutrition and public health. In the coming decades, the programme will continue to focus on inclusivity, adaptability, and vision, demonstrating that investing in people is the most powerful lever for advancing nutrition and public health, in line with the words of the new IUNS President, Professor Hyun-Sook Kim:

*To young nutrition scientists:*

*You are not just the future - you are already shaping the present. Your curiosity, your compassion, and your creativity will define how our nutrition science serves humanity. Remember, the leadership is not about titles; it is about purposes. It is about ‘Caring Deeply and Acting Boldly’’ I strongly believe you have that power. Let us continue our shared global journey together.*
